# Enzyme production by filamentous fungi: analysis of the secretome of *Trichoderma reesei *grown on unconventional carbon source

**DOI:** 10.1186/1475-2859-10-68

**Published:** 2011-08-23

**Authors:** He Jun, Thomas Kieselbach, Leif J Jönsson

**Affiliations:** 1 Department of Chemistry, Umeå University, Umeå, Sweden; 2 Research Institute of Animal Nutrition, Sichuan Agricultural University, Ya'an, Sichuan 625014, P. R. China

## Abstract

**Background:**

Spent hydrolysates from bioethanolic fermentation processes based on agricultural residues have potential as an abundant and inexpensive source of pentose sugars and acids that could serve as nutrients for industrial enzyme-producing microorganisms, especially filamentous fungi. However, the enzyme mixtures produced in such media are poorly defined. In this study, the secretome of *Trichoderma reesei *Rut C-30 grown either on a spent hydrolysate model medium (SHMM) or on a lactose-based standard medium (LBSM) was explored using proteomics.

**Results:**

Our results show that both the SHMM and LBSM serve as excellent growth media for *T. reesei *Rut C-30. In total, 52 protein spots on 2-D gels were identified by using matrix-assisted laser desorption/ionization mass spectrometry (MALDI-MS) and electrospray ionization liquid chromatography tandem mass spectrometry (ESI-LC MS/MS). As expected, a considerable number of the identified proteins were related to the degradation of lignocellulosic biomass. The enzyme production profiles in the two media were similar, but β-glucosidase and β-galactosidase were only produced in LBSM. The main cellobiohydrolases (Cel7A/Cel6A) and endoglucanases (Cel7B/Cel5A) were identified in both media and the cellobiohydrolases, i.e. Cel7A and Cel6A, were the most abundant cellulolytic enzymes. Moreover, both media can also serve as a potent inducer of xylanolytic enzymes. Several key enzymes involved in sugar assimilation and regulation of cellulase formation were identified, and were found to be differentially expressed in the two growth media.

**Conclusions:**

This study not only provides a catalogue of the prevalent proteins secreted by *T. reesei *in the two media, but the results also suggest that production of hydrolytic enzymes using unconventional carbon sources, such as components in spent hydrolysates, deserves further attention in the future.

## Background

Lignocellulose is the most abundant renewable resource on earth. It can be hydrolyzed to sugars, which then can be fermented to ethanol or other commodities by various microorganisms [1]. Although replacement of gasoline with lignocellulosic ethanol has attracted considerable research interest in recent years, the high cost of hydrolyzing lignocellulosic polysaccharides to fermentable sugars remains a major obstacle for efficient bioethanol production [2]. As the costs for cellulolytic enzymes contribute substantially to the price of bioethanol, less expensive sources of these enzymes would be preferred [3]. For this purpose, considerable research efforts have been focused on producing more efficient enzymes from cheaper growth media using various microorganisms [4-8].

Agricultural residues, such as sugarcane bagasse, straw and corn stover, are among the most attractive feedstocks for production of cellulosic ethanol, and spent hydrolysates (stillages) from a cellulose-to-ethanol process would be a major residue containing large amounts of the pentose sugars xylose and arabinose, which are not normally utilized by the industrially preferred yeast *Saccharomyces cerevisiae*. However, these unfermented sugars are likely to serve as an inexpensive nutrient source for industrial enzyme-producing microorganisms, especially metabolically diverse filamentous fungi. For instance, the suitability of spent sugarcane bagasse hydrolysate as a growth medium for a Cel7B-expressing *Aspergillus niger *was clearly reflected by a high microbial biomass production, high levels of heterologous protein production, and high levels of endoglucanase activity [5].

Filamentous fungi are the preferred source of industrial enzymes because of their excellent capacity for extracellular protein production. With regard to industrial production of cellulolytic enzymes, the soft-rot fungus *Trichoderma reesei *(syn. *Hypocrea jecorina*) is known to secrete large amounts of cellulases and can also utilize a broad range of carbon sources including pentose sugars [9,10]. It is possible that large-scale commercial production of cellulolytic enzymes could benefit from the utilization of spent hydrolysates or similar residues as nutrient source for *T. reesei*. Previous studies have indicated that *T. reesei *produces a relatively smaller number of cellulases and hemicellulases compared to other plant-cell-wall-degrading fungi, and only ten cellulase genes and sixteen hemicellulase genes were identified through sequencing the whole *T. reesei *genome [11]. The genes related to degradation of lignocellulosic biomass are potentially transcribed during the cultivation of the fungus, but the relative proportions of the expressed proteins may vary widely depending on the growth medium and the cultivation conditions [12]. For instance, the extracellular cellulolytic system of *T. reesei *in response to 1 mM sophorose is composed of 85% cellobiohydrolases, 15% of endoglucanases and only 1% of β-glucosidase [13], which is quite different from the enzyme mixtures produced in sorbose- or lactose-based media [14]. Although it would be beneficial if the spent hydrolysates can serve as an inexpensive nutrient source for *T. reesei*, the enzyme mixtures produced in such media are not well characterized with respect to protein identification and quantification.

The aim of this study was to perform a proteomics study of the proteins secreted by *T. reesei *Rut C-30, which is a strain already used in industrial scale. The fungus was grown on a spent hydrolysate model medium (SHMM) or on a lactose-based standard medium (LBSM). SHMM was used in order to have well-defined conditions for cultivations and subsequent proteomics experiments. The major nutrient components of the SHMM, namely monosaccharides (xylose, arabinose, and galactose) and acetic acid, were chosen on the basis of chemical analyses performed on an authentic spent hydrolysate [5]. This study provides the mapping of the proteins secreted by *T. reesei *in SHMM and LBSM, and offers a basis for further exploration of enzyme production using unconventional carbon sources.

## Results

### Cultivation of *T. reesei *Rut C-30 in SHMM and LBSM

Since the precise composition of spent hydrolysates is complex and may differ depending upon pretreatment and other factors, a model medium with well defined content was used to carry out the investigation. Based on a spent hydrolysate from sugarcane bagasse [5], D-xylose was the major carbon source in the model medium. As shown in Table 1, all three monosaccharides were utilized by *T. reesei*. However, some sugar still remained in the culture medium when sample for secretome analysis was taken, so the fungal cultures where not starved with respect to carbon source. The results shown in Table 1 also show that *T. reesei *was able to consume or convert acetic acid. Both the LBSM and the SHMM serve as an excellent growth medium for *T. reesei*. However, the microbial biomass production after 7 days of cultivation was higher in the LBSM (Table 2).

**Table 1:** Major nutrient components (carbon sources) of SHMM

Carbon source (g/L)	Initial content	Final content^a^
Xylose	9.5	3.1
Arabinose	0.5	0.15
Galactose	0.5	0.22
Acetic acid	0.9	< 0.1

^a ^Determined at the end of the cultivation.

**Table 2:** Growth and enzyme production by *T. reesei *Rut C-30 cultivated in SHMM or LBSM

Item	LBSM	SHMM
Biomass (DW g/L)	6.18 ± 0.41	4.18 ± 0.37
Protein content (mg/mL)	2.18 ± 0.17	3.36 ± 0.26
FPase^a ^(μmol glucose/min/mg protein)	0.49 ± 0.05	0.29 ± 0.04
CMCase^b ^(μmol glucose/min/mg protein)	4.33 ± 0.41	1.66 ± 0.20
Xylanase (μmol xylose/min/mg protein)	5.35 ± 0.61	4.52 ± 0.52

^a ^FPase represents filter paper activity.

^b ^CMCase represents activity on carboxymethyl cellulose (CMC).

### Extracellular enzyme production by *T. reesei *Rut C-30

As shown in Table 2, cultivation of *T. reesei *Rut C-30 in SHMM resulted in a high concentration of total protein in the culture supernatant. As compared to the SHMM, cultivation of the fungus in LBSM significantly elevated the FPase and CMCase activity (Table 2). Xylanolytic enzymes were produced in both media, and the total xylanase activity produced in LBSM was 18% higher than in the SHMM (Table 2).

### 2-DE mapping of the *T. reesei *Rut C-30 secretome

**Figure 1:**
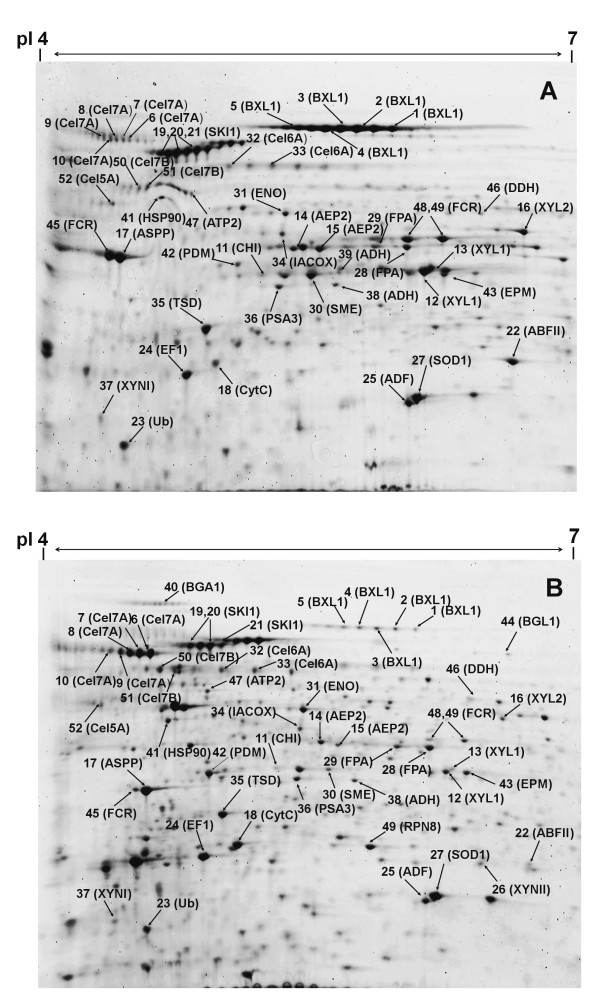
**2-DE analysis of secreted proteins by *T. reesei Rut *C-30 grown on SHMM (A) or on LBSM (B)**. The identified protein spots are labeled by the protein abbreviations given in Table 3.

**Figure 2:**
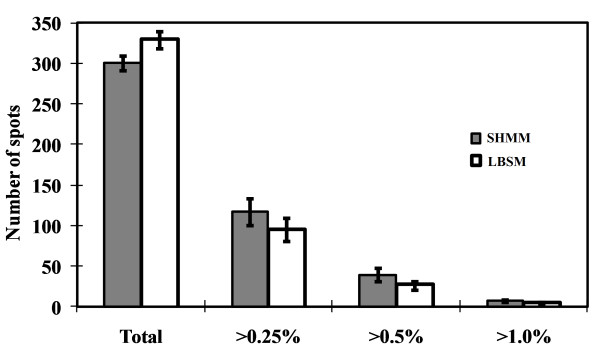
**Spot volume distribution of secreted proteins by *T. reesei *Rut C-30 grown on SHMM or on LBSM**. Standard deviations were calculated from three replicates.

Two-dimensional gel electrophoresis (2-DE) is considered as a powerful tool to separate and visualize hundreds of proteins at a time, which in combination with MS offers a way to identify them. In this investigation, the total extracellular protein from the culture supernatants was separated by 2-DE. Preliminary investigations using pH 3 to 10 IPG strips indicated that most of the proteins secreted by *T. reesei *Rut C-30 had p*I*s below 7. To improve the resolution and facilitate the quantification of separated protein spots, IPG strips with a pH range from 4 to 7 were selected for further IEF experiments. The protein maps are shown in Figure 1. The distribution of the protein spots indicates that most of the secreted proteins have an isoelectric point below 6 and a molecular mass above 34 kDa. We also quantified the number of protein spots using the ImageMaster II software, and more than three hundred protein spots were detected on the 2-D gels after fluorescence staining using SYPRO Ruby. As shown in Figure 2, more protein spots (total numbers) were detected in LBSM than in SHMM. However, the difference was not statistically significant. Among these protein spots, 52 were identified by MALDI-MS (matrix-assisted laser desorption/ionization mass spectrometry) and ESI-LC MS/MS (electrospray ionization liquid chromatography tandem mass spectrometry). As expected, a considerable number of the identified proteins were related to the degradation of lignocellulosic biomass (Table 3).

**Table 3:** Identification by MALDI-MS and ESI-LC MS/MS of proteins secreted by *T. reesei *Rut C-30 grown in SHMM and LBSM

Num	ProtID^a^	Protein name	Precursor mass (kDa)	p*I*^b^	**Mascot score**^**c**^	Peptides matched	%Sequence coverage	Sig^d^
1	121127	β-Xylosidase (BXL1)	87.1	5.9	110 *	14	29.0	Y
2	121127	β-Xylosidase (BXL1)	87.1	5.8	134 *	18	31.5	Y
3	121127	β-Xylosidase (BXL1)	87.1	5.7	133 *	16	26.0	Y
4	121127	β-Xylosidase (BXL1)	87.1	5.6	127 *	17	29.5	Y
5	121127	β-Xylosidase (BXL1)	87.1	5.5	102 *	16	28.6	Y
6	123989	Cellobiohydrolase I (Cel7A)	54.1	4.4	64 *	6	16.7	Y
7	123989	Cellobiohydrolase I (Cel7A)	54.1	4.6	62 *	6	16.7	Y
8	123989	Cellobiohydrolase I (Cel7A)	55.4	4.5	73*	5	13	Y
9	123989	Cellobiohydrolase I (Cel7A)	55.4	4.5	73*	5	13	Y
10	123989	Cellobiohydrolase I (Cel7A)	55.4	4.4	73*	5	13	Y
11	81598	Chitinase (CHI)	44.5	5.3	464^#^	9	18	Y
12	107776	D-Xylose reductase (XYL1)	36.5	6.1	99 *	10	44.4	N
13	107776	D-Xylose reductase (XYL1)	36.5	6.1	606^#^	12	39	N
14	121661	Aldose 1-epimerase 2 (AEP2)	37.1	5.3	194 *	18	62.9	N
15	121661	Aldose 1-epimerase 2 (AEP2)	37.1	5.4	136 *	17	56.4	N
16	22426	D-xylose dehydrogenase (XYL2)	41.5	6.7	241 *	21	67	Y
17	77579	Aspartyl protease (ASPP)	42.4	4.5	118 *	11	25	Y
18	58493	Cytochrome c oxidase, subunit Vb (CytC)	16.6	5.2	175^#^	4	20	N
19	51365	Subtilisin kexin isozyme-1 (SKI1)	93.3	4.6	111 *	10	17.9	Y
20	51365	Subtilisin kexin isozyme-1 (SKI1)	93.3	4.7	143 *	12	22.7	Y
21	51365	Subtilisin kexin isozyme-1 (SKI1)	93.3	4.8	142 *	12	21.2	Y
22	120911	Predicted arabinofuranosidase (ABFII)	36.3	6.7	379^#^	14	31	Y
23	42919	Ubiquitin fusion protein (Ub)	8.3	4.5	89^#^	3	41	N
24	123902	Translation elongation factor 1 (EF1)	45.9	4.6	109 *	14	37	Y
25	73967	Actin depolymerizing factor (ADF)	17.4	6.0	108 *	7	53	N
26	81586	Xylanase II (XYN II)	18.8	6.5	104 *	8	47	Y
27	123029	Copper/Zinc superoxide dismutase (SOD1)	15.8	6.1	80 *	5	55	Y
28	23200	Fructose 1,6-bisphosphate aldolase (FPA)	39.4	6.0	142 *	13	45	N
29	23200	Fructose 1,6-bisphosphate aldolase (FPA)	39.4	5.9	99*	16	50.8	N
30	2433	7alpha-Cephem-methoxylase (SME)	32.1	5.4	149 *	20	84	N
31	120568	Enolase (ENO)	47.3	5.2	143 *	15	43	N
32	72567	Cellobiohydrolase II (Cel6A)	50.3	5.2	100 *	9	27	Y
33	72567	Cellobiohydrolase II (Cel6A)	50.3	5.4	107*	10	27	Y
34	73631	Isoamyl alcohol oxidase (IACOX)	60.9	5.2	169 *	17	32	Y
35	123026	Transaldolase (TSD)	35.6	5.1	78 *	9	29	N
36	73564	20S proteasome, subunit (PSA3)	31.7	5.1	99 *	10	34	N
37	74223	Xylanase I (XYN I)	24.6	4.4	86^#^	5	49.6	Y
38	78683	Aldehyde dehydrogenase (ADH)	53.7	5.5	250^#^	6	14	N
39	78683	Aldehyde dehydrogenase (ADH)	53.7	5.5	334^#^	5	12	N
40	73842	Predicted beta-galactosidase (BGA1)	97.5	5.1	85 *	10	39	Y
41	123114	Similar to Hsp90	78.5	4.7	87^#^	2	5	Y
42	105808	Peptidase M (PDM)	33.9	4.9	79 *	5	22	Y
43	107639	Predicted epimerase (EPM)	35.1	6.2	183 *	17	72	Y
44	76672	Beta-glucosidase (BGL1)	78.7	6.4	196 *	20	49	Y
45	109282	Flavonol/cinnamoyl-CoA reductase (FCR)	39.3	4.5	90 *	9	33.0	N
46	22426	Dimeric dihydrodiol dehydrogenase (DDH)	41.5	6.5	241 *	21	67	Y
47	123071	ATP synthase beta chain (ATP2)	54.8	5.3	212 *	23	62	N
48	79324	Flavonol/cinnamoyl-CoA reductase (FCR)	38.2	6.1	137 *	15	42.9	N
49	79324	Flavonol/cinnamoyl-CoA reductase (FCR)	38.2	6.2	156 *	12	35.2	N
50	122081	Endoglucanase I (Cel7B)	48.2	4.7	402^#^	13	16.3	Y
51	122081	Endoglucanase I (Cel7B)	48.2	4.8	106^#^	4	9.2	Y
52	120312	Endoglucanase II (Cel5A)	44.1	5.8	219^#^	5	18.4	Y

^a ^From *T. reesei *genome database: http://genome.jgi-psf.org/Trire2/Trire2.home.html.

^b ^*p*Is determined by 2-DE.

^c ^Analyzed by MALDI-MS (*) or LC-MS/MS (^#^)

^d ^With (Y) or without (N) predicted signal sequence for exporting.

The enzyme production profiles in the two growth media were qualitatively similar although some proteins, such as β-glucosidase (BGL1) and β-galactosidase (BGA1), were only detected in samples from the LBSM. The cellobiohydrolases Cel7A and Cel6A were the most abundant of the cellulolytic enzymes secreted by *T. reesei *(Figure 1). Two out of five known endoglucanases (Cel7B and Cel5A) were identified in both media. However, as compared to the cellobiohydrolases, the endoglucanases were produced in low amounts. In addition, some major components of the hemicellulolytic system of *T. reesei*, such as β-xylosidase (BXL1), various xylanases, and arabinofuranosidase (ABFII), were identified on the 2-D gels. Apart from BXL1, the hemicellulolytic enzymes were expressed in low amounts. Moreover, several key enzymes involved in sugar assimilation and regulation of cellulase formation were identified (e.g. AEP2 and XYL1) (Table 3).

### Quantification and comparison of the protein produced in different media

**Figure 3:**
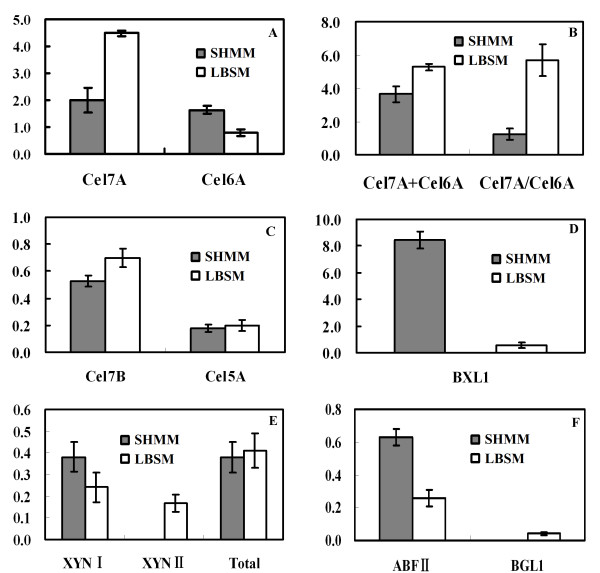
**Comparative analysis of the secretome of *T. reesei *Rut C-30 grown on SHMM or on LBSM**. A: Volume of Cel7A and Cel6A; B: Volume of total cellobiohydrolases and Cel7A-to-Cel6A ratio; C: Volume of Cel7B and Cel5A; D: Volume of β-xylosidase; E: Volume of xylanases; F: Volume of other identified lignocellulosic-biomass-degrading enzymes.

The relative amount for each protein spot (% of total spots volume) was quantified using the ImageMaster II software. The spot numbers detected on the two protein maps were similar, especially in minor spots corresponding to less than 0.5% of total spots volume (Figure 2). *T. reesei *Rut C-30 produced more Cel6A in the SHMM than in the LBSM (Figure 3A). Nevertheless, the standard medium contained 44% higher amount of cellobiohydrolase (Cel7A + Cel6A) than the SHMM (5.3% vs. 3.7%). This is related to the higher Cel7A level in the LBSM than in the SHMM (4.5% vs. 2.0%). As a consequence, the ratio of Cel7A to Cel6A is much higher in the standard medium (Figure 3B). *T. reesei *produced slightly more endoglucanases (Cel7B + Cel5A) in the LBSM than in the SHMM (0.7% vs. 0.8%). However, the amount of BXL1 was 13.6-fold higher in the SHMM than in the standard medium (Figure 3D).

**Figure 4:**
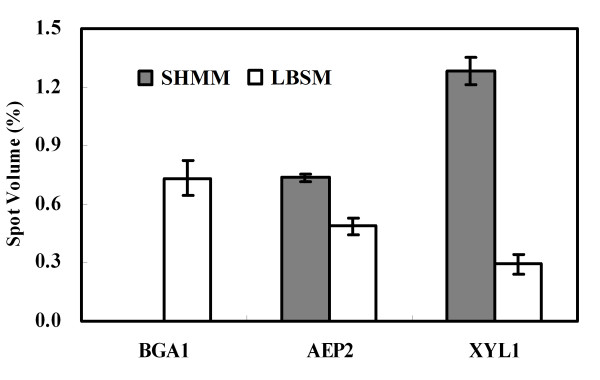
**Spot volume quantification of proteins involved in sugar assimilation and regulation of enzyme formation**. *T. reesei *Rut C-30 was cultivated in SHMM or LBSM.

Two out of four known *T. reesei *xylanases were identified. The fungus produced more xylanase I (XYN I) in the SHMM than in the LBSM, whereas xylanase II (XYN II) was found almost exclusively in the LBSM (Figure 3E). No significant difference was observed with respect to the total amount of xylanase (XYN I + XYN II) in the two growth media (Figure 3E). The β-glucosidase (BGL1) was only produced in the LBSM (Figure 3F). However, *T. reesei *Rut C-30 produced more ABFII in the SHMM (Figure 3F). Proteins involved in sugar assimilation and cellulase regulation were found to be differentially expressed in the two growth media. As shown in Figure 4, β-galactosidase (BGA1) was only produced in the standard medium, while aldose 1-epimerase 2 (AEP2) and xylose reductase (XYL1) were found in both media. The relative amount of AEP2 and XYL1 was, however, significantly higher in the SHMM than in the LBSM (Figure 4).

## Discussion

It is a well known fact that the production of the main *T. reesei *enzymes related to degradation of lignocellulosic biomass is transcriptionally regulated and carbon source dependent [14-16]. The enzyme formation can be induced by various carbohydrates and their derivatives, including lactose, sophorose, xylobiose, D-xylose, and L-sorbose [14,17]. However, the range of technically applicable substrates is still limited since most of the above-mentioned carbon sources are too expensive for industrial fermentations. Spent hydrolysates, which would be one of the main residues from production of second-generation ethanol, would contain various monosaccharides, predominantly xylose, and other organic substances [5]. Although, the suitability of spent hydrolysate as a growth medium for a Cel7B-expressing *Aspergillus niger *has already been investigated [5], the enzyme mixtures produced by filamentous fungi growing in such a medium are still poorly characterized. In the present study, the composition of the secretome of *T. reesei *Rut C-30 grown on a spent hydrolysate model medium was explored using proteomics and compared to a conventional lactose-based medium. We found that the SHMM can serve as an excellent growth substrate for *T. reesei *Rut C-30. However, the reference fermentation with standard medium containing 10 g/L lactose resulted in higher microbial biomass production and higher total cellulase activity (Table 2). These results suggest that the cellulase production by *T. reesei *is carbon-source dependent, and that lactose, which is a commonly used carbon source in industrial production media, not only promotes good growth but also efficiently induces the expression of cellulolytic genes [18,19]. Differences in microbial biomass production and cellulase activity could tentatively also arise as a consequence of other differences between the two growth media than the carbon source. However, there are studies indicating that the induction of cellulolytic enzymes is more dependent on the carbon source than on other components in the medium [15,20].

Our study shows that 0.9 g/L acetic acid, which is a commonly occurring inhibitor in lignocellulose hydrolysates, did not prevent growth of *T. reesei*, which instead converted most of it. The potentially inhibitory furan aldehydes furfural and HMF (5-hydroxymethylfurfural) were not included in the model medium. In an authentic spent hydrolysate, the furan aldehydes would to a large extent have been reduced by yeast to the non-inhibitory corresponding alcohols, as was observed by Alriksson et al. [5]. Therefore, inclusion of furan aldehydes in the model medium was not considered to be relevant.

*T. reesei *is an extraordinarily efficient producer of cellulolytic enzymes [14,19] although it has a relatively small number of cellulases-encoding genes for being a plant-cell-wall-degrading fungus [11]. In the present study, 52 protein spots were identified by MALDI-MS and ESI-LC MS/MS. As expected, most of the identified proteins contain a signal peptide (sequence) responsible for exporting the protein to the extracellular space (Table 3). *T. reesei *expresses only two cellobiohydrolases (Cel7A and Cel6A; EC 3.2.1.91), enzymes that catalyze the release of cellobiose from reducing or non-reducing end of cellulose, and five endoglucanases (Cel7B, Cel5A, Cel12A, Cel61A, and Cel45A; EC 3.2.1.21), enzymes that attack cellulose in an endo-acting manner [21]. In the present study, two endoglucanases, Cel7B and Cel5A, were detected on the 2-D gels. The fact that the two cellobiohydrolases (Cel7A and Cel6A) were identified as occurring in relatively large amounts both in the SHMM and in the LBSM is consistent with previous findings that Cel7A and Cel6A are the two most abundantly secreted enzymes and account for 70 to 80% of the total cellulase in *T. reesei *[21,22]. The Cel7A/Cel6A ratio was much higher in the LBSM than in the SHMM. This finding is surprising considering the widespread hypothesis that the expression of Cel7A and Cel6A is co-regulated [23]. Our results are, however, consistent with the conception of the carbon-source-dependent nature of the cellulolytic system of *T. reesei*.

β-Glucosidase (EC 3.2.1.21) catalyzes the hydrolysis of cellobiose to glucose [24]. Although the molecular properties of this enzyme are well established, less is known about the regulation of its formation. Sophorose, one of the most potent inducers of *T. reesei *cellulases, does not induce β-glucosidases and, in high concentrations, even represses its formation [25]. Like sophorose, the monosaccharides in the SHMM (xylose, arabinose, and galactose) were unable to induce β-glucosidases. The main β-glucosidase, BGL1, was, however, identified in the LBSM. The presence of β-glucosidase may contribute to a higher cellulase activity in this medium since there are some reports suggesting that BGL1 is involved in the formation of cellulase inducers [26-28].

We also identified several major components of the hemicellulolytic system of *T. reesei *(Table 3). β-Xylosidase, BXL1, which is required for complete degradation of xylan to xylose, is the limiting enzyme in xylan hydrolysis by *T. reesei *[29]. In the present study, the level of BXL1 showed a 13.6-fold increase in the SHMM. This result agrees well with previous observations that formation of BXL1 by *T. reesei *can be induced very specifically by xylose, and that other compounds that yield xylose upon breakdown (i.e. xylan and xylobiose) also cause induction of this enzyme [30]. Similarly, formation of xylanases is also promoted by xylan or cellulose-degradation products [31,32]. Although, lactose has been considered as one of the most potent inducers of xylanases [33], no significant difference was observed with respect to the total level of xylanases (XYN I + XYN II) in the two growth media. Moreover, two other *T. reesei *xylanases (XYN III and XYN IV) were not detected, which can be due to that they were produced only in low amounts and that they have p*I*s above 7 [34-36]. Production of xylanolytic enzymes may also be affected by pH, since XYN I is expressed at low pH, and XYN II and XYN III at higher pH [37,38]. In the present study, the final pH in the SHMM was slightly lower than in the LBSM (6.9 vs. 7.2). XYN II was detected only in LBSM, whereas XYN I was detected in both media. In *T. reesei*, there are some parallels between the regulation of xylanolytic enzymes and the regulation of the formation of cellulases by cellulose and cellulose-degradation products [24,25], which suggests the potential of regulation of the expression of depolymerizing enzymes through various control mechanisms.

With the use of fluorescence staining, over three hundred protein spots were detected on 2-D gels (Figure 2), which is higher than the number reported by Herpoel-Gimbert et al., despite that they used a lower threshold in their study [22]. Thus, more protein spots were subsequently selected for MS identification. Analyses of these proteins not only led to the identification of the main lignocellulolytic enzymes (such as Cel7A and Cel6A), but also led to the identification of several key enzymes (such as BGA1 and AEP2) that are involved in sugar utilization and regulation of cellulase formation [39,40]. The β-galactosidase, BGA1, which is a hydrolase belonging to glycosyl hydrolase family 35, catalyzes the hydrolysis of β-galactosides into monosaccharides. More recently, it has been identified as a critical factor for the production of *T. reesei *cellulases when the fungus is grown on lactose-based medium [41]. In the present study, BGA1 was only identified in the LBSM, but not in the SHMM. Although, L-arabinose can also act as an efficient inducer of BGA1 [41], it accounts only for a small part of the monosaccharides in the SHMM.

Aldose 1-epimerase 2 (AEP2) and xylose reductase (XYL1) are important enzymes in lactose and xylose metabolism, respectively [42,43]. Both of them were identified on the 2-D gels. This is surprising, since AEP2 and XYL1 have been thought to be intracellular proteins [44,45]. A previous study indicated that cellulase induction in *T. reesei *by lactose requires the β-anomer of D-galactose, and the decrease of mutarotase activity (e. g. AEP) during growth on lactose is an important factor for enzyme production [44]. The present results agree well with this report since the *T. reesei *produces less AEP2 in the LBSM than in the SHMM (Figure 4). The XYL1 is also important for the production of cellulases on lactose since deletion of the *XYL1 *gene significantly reduced the formation of BGA1 and Cel7A in *T. reesei *[46]. In the present study, *T. reesei *produced considerably more XYL1 in the SHMM than in the LBSM (Figure 4), which agrees well with previous findings that the D-xylose is a stronger XYL1 inducer than lactose [46,47]. Since the *T. reesei *produced less Cel7A and BGA1 in SHMM than in LBSM, our results suggest that XYL1-dependent induction of cellulases on lactose only serves as a subordinate regulation pathway in *T. reesei*, and that very high XYL1 levels, such as in the SHMM, may even be associated with less efficient induction of cellulases. It is noteworthy in this context that XYL1 has been identified as a major aldose reductase in the second pathway for D-galactose catabolism [46], and that both D-galactose and D-galactose-1-phosphate can act as inducers of cellulase [48].

## Conclusions

The SHMM was found to serve as an excellent growth medium for *T. reesei *Rut C-30 and efficiently induced the production of cellulolytic enzymes. With this medium, the fungus not only produces considerable amount of cellulases, but also high levels of xylanolytic enzymes. The results suggest that spent hydrolysates are suitable as an inexpensive nutrient source also for other industrially important enzyme-producing microbes than the well-studied *A. niger *[5]. Moreover, the carbon source-dependent induction of cellulolytic enzymes in *T. reesei *may result in part from the altered expression levels of several important metabolic enzymes, such as the AEP2 and XYL1. This study not only provides a catalogue of the prevalent proteins secreted by *T. reesei *in SHMM and LBSM, but the results also suggest that production of hydrolytic enzymes using unconventional carbon sources deserves further attention in the future, for example by studying the performance of the fungus in fed-batch cultures based on spent hydrolysates.

## Methods

### Strain and culture conditions

*Trichoderma reesei *Rut C-30 was maintained on Potato Dextrose Agar (PDA) plates incubated at 30°C. Before inoculation, the spores were resuspended in sterile water to achieve a final concentration of 1 × 10^6 ^spores/mL. For the experiments with SHMM, each of four 100 mL Erlenmeyer flasks were filled with 48 mL of a carbon source mixture (9.5 g/L D-xylose, 0.5 g/L L-arabinose, 0.5 g/L galactose, 0.9 g/L acetic acid), 1 mL of nutrient solution [25 g/L (NH_4_)_2_HPO_4_, 1.25 g/L MgSO_4_·7H_2_O, 69 g/L NaH_2_PO_4_·H_2_O, and 50 g/L yeast extract], and 0.05 mL of trace element solution (0.22 g/L ZnSO_4_·7H_2_O, 0.11 g/L H_3_BO_3_, 0.05 g/L MnCl_2_·4H_2_O, 0.05 g/L FeSO_4_·7H_2_O, 0.017 g/L CoCl_2_·6H_2_O, 0.016 g/L CuSO_4_·5H_2_O, 0.015 g/L Na_2_MoO_4_·2H_2_O, and 0.5 g/L EDTA). The components of the SHMM medium were based on the study by Alriksson et al. [5]. The initial pH of this and other media used for cultivation of *T. reesei *was approximately 6.0. For the experiments with LBSM, each of four 100 mL Erlenmeyer flasks were filled with 49.05 mL of a lactose-based standard medium containing 10 g/L lactose [14]. All flasks were inoculated with 0.95 mL of the *T. reesei *Rut C-30 spore solution with a concentration of 1 × 10^6 ^spores/mL. Then, they were closed with cotton plugs and were incubated for 7 days in an incubator (AG CH-4103, Infors, Bottmingen, Switzerland) with shaking at 30°C and 150 rpm. The microbial biomass production was determined at the end of the experiment.

### Sample collection and protein extract preparation

At the end of cultivation, samples (culture supernatants) were harvested by centrifugation for 15 min at 12,000 g and 4°C. The proteins in the supernatant were precipitated and purified by using a 2-D clean-up kit (GE Healthcare, Uppsala, Sweden). The purified protein sample was dissolved in rehydration solution [4% CHAPS (3-[(3-cholamidopropyl) dimethylammonio]-1-propanesulfonate), 8 M urea, 0.002% bromophenol blue] supplemented with 2% (v/v) 4-7 IPG buffer (GE Healthcare) and 2.8 mg/mL dithiothreitol. Total protein concentration was determined using the 2-D Quant kit (GE Healthcare). Aliquots of extracellular protein samples were stored at -80°C before proteomic assays.

### Protein separation by two-dimensional gel electrophoresis (2-DE)

Regardless of the initial protein concentration in the culture supernatant, the same amount of protein (200 μg) was used for each 2-D gel. Immobiline DryStrips (18 cm, pH 4-7, GE Healthcare) were rehydrated overnight at room temperature with 200 μg of protein dissolved in rehydration solution (about 350 μL). Isoelectric focusing (IEF) was performed with a Multiphor II system (GE Healthcare) at 20°C with a 3-phase gradient program: 500 V for 0.25 kVh, 3500 V for 5.25 kVh, and 3500 V for 28 kVh. Following IEF, each strip was equilibrated for 12 min in 10 mL of SDS equilibration buffer [50 mM Tris-HCl, 6 M urea, 30% (v/v) glycerol, 2% (w/v) SDS, 0.002% bromophenol blue] containing 1% (w/v) dithiothreitol. A second equilibration step was then performed with 2.5% (w/v) iodoacetamide added to the SDS equilibration buffer. The strips were then loaded onto 12.5% homogeneous ExcelGels (GE Healthcare). The second dimension of the separation was performed using the Multiphor II system operating at 15°C and with the following 2-phase gradient program: 120 V for 0.5 h and 600 V for 1 h. After electrophoresis, the gels were either stained with SYPRO Ruby Protein Gel Stain (Invitrogen, Carlsbad, CA) (for comparative analysis experiments) or with Coomassie Brilliant Blue (CBB) R250 (for spot-picking experiments).

### Image analysis

For comparative analysis, each culture sample was independently prepared and used in 2-DE analyses performed in triplicates. Gels were scanned on a Typhoon 9400 scanner (GE Healthcare). Images were analyzed using ImageMaster II software (GE Healthcare). After automatic spot detection, artifacts, such as dust on gels, were manually removed, and the weaker spots (< 0.1% of the whole gel volume) were eliminated. The remaining spots were then automatically linked to reference spots on a synthetic reference gel to allow comparison of samples.

### Preparation of in-gel digests

For analysis by mass spectrometry, protein spots were picked from the 2-D gels using an Ettan Spotpicking Station (GE Healthcare) and destained three times using a fresh solution of 20 mM ammonium bicarbonate containing 35% (v/v) acetonitrile (ACN). Subsequently, the gel pieces were dried by two washes using 100% neat acetonitrile and re-hydrated on ice using a solution of sequencing-grade modified trypsin (Promega, Madison, WI) in 20 mM ammonium bicarbonate. The trypsin concentration depended on the intensity of the spots and was 2 to 3 ng/μL. The re-hydrated gel samples were incubated in 37°C for overnight digestion.

### Mass spectrometry

MALDI-MS spectra for peptides were acquired using a Voyager DE-STR mass spectrometer (AB SIEX, Stockholm, Sweden) as described by Yao et al. [49]. LC-MS/MS combined with ESI-ion-trap MS was performed using an HCT-Ultra ETD II mass spectrometer from Bruker (Bremen, Germany) linked to an Easy-nLC system from Proxeon (Odense, Denmark). Spectra were acquired using the enhanced scanning mode covering a mass range from *m/z *300 to *m/z *1300. The LC separation of peptides was performed using a 5 μm C18 column (375 μm OD/75 μm ID×10 cm) from NanoSeparations (Nieuwkoop, The Netherlands) and a 30 min gradient ranging from 0 to 60 percent of acetonitrile. The flow rate was 300 nL min^-1^. Database searches using the peak list files of the processed mass spectra were performed using an in-house license of Mascot (http://www.matrixscience.com) and the *T. reesei *database of the JGI (http://www.jgi.doe.gov/sequencing/why/2998.html). As for MALDI-MS spectra, a mass error of 50 ppm and one missed cleavage site were permitted. In addition, variable modifications allowed included methionine oxidation and carbamidomethylation of cysteine residues. As for the LC-MS/MS data, a mass error of 0.3 Da was allowed for both the MS and MS/MS mode and variable modifications were set as for the database searches with the MALDI-MS data.

### Enzymatic assays

Enzymatic activities were measured in culture supernatants obtained after centrifugation. Overall cellulase activity of the samples was determined as filter paper activity (FPase) using Whatman no.1 filter paper strips as the substrate [50]. Endoglucanase activity was measured as CMCase activity with CMC (carboxymethyl cellulose) dissolved in 50 mM citrate buffer (pH 5.0) as the substrate. The assay was performed for 30 min at 50°C. Xylanase activity was assayed by the method described by Bailey et al. [51]. Oat spelt xylan (Sigma-Aldrich, St. Louis, MO) was used as the substrate. The amount of released sugar was assayed via the dinitrosalicylic acid (DNS) method using glucose or xylose as the standard [52].

### Biomass measurement and chemical analysis

To determine the microbial biomass production, Miracloth (Calbiochem, Merck KGaA, Darmstadt, Germany) was used and the dry weight (DW) of *T. reesei *was determined by using the procedure described by Alriksson et al. [5]. The monosaccharide content in the SBH model medium remaining after the cultivation was analyzed by MoRe Research (Örnsköldsvik, Sweden).

## Competing interests

The authors declare that they have no competing interests.

## Authors' contributions

HJ carried out the cultivation of the fungus, the enzymatic assays, the sample preparation and the 2-DE analyses, and drafted the manuscript. TK carried out in-gel digestions and mass- spectrometric analyses. LJJ conceived and organized the study and helped to draft the manuscript. All authors read and approved the final manuscript.
